# Cutaneous Expression of A Disintegrin-like and Metalloprotease domain containing Thrombospondin Type 1 motif-like 5 (ADAMTSL5) in Psoriasis goes beyond Melanocytes

**DOI:** 10.4172/2376-0427.1000244

**Published:** 2016-09-19

**Authors:** Kathleen M Bonifacio, Norma Kunjravia, James G Krueger, Judilyn Fuentes-Duculan

**Affiliations:** Laboratory for Investigative Dermatology, The Rockefeller University, New York, USA

**Keywords:** Psoriasis, ADAMTSL5, Melanocytes, Keratinocytes, T-Cells, Dendritic cells

## Abstract

A Disintegrin-like and Metalloprotease domain containing Thrombospondin type 1 motif-like 5 (ADAMTSL5) is a melanocyte-derived protein that has recently been implicated as an activating antigen for IL-17-producing T cells in psoriasis. There is a potential disconnect between the basal location of the melanocytes in the epidermis and the fact that T-cell infiltrates are seen mostly scattered in the epidermis with very large infiltrates in the dermis. Thus, we hypothesized that ADAMTSL5 may be expressed in other cells aside from melanocytes in skin. To further investigate the cutaneous expression of ADAMTSL5, we performed immunohistochemistry staining on lesional and nonlesional skin biopsies from psoriasis patients using three different commercially available antibodies. We confirmed that all ADAMTSL5 antibodies reacted with epidermal melanocytes. However, we also observed a strong expression of this protein in keratinocytes throughout the epidermis, and scattered expression in some dermal blood vessels and other perivascular dermal cells. Overall, the pattern of ADAMTSL5 expression is very similar to the infiltrating pattern of T-cells and dendritic cells in psoriasis lesions.

## Introduction

Psoriasis vulgaris is a chronic inflammatory skin disease characterized by thick scaly plaques affecting about 2–3% of Caucasians [1]. Histologic hallmarks of psoriasis include hyperproliferation of keratinocytes (acanthosis) in the epidermis, immature cells in the stratum corneum (parakeratosis), thickening of the stratum corneum (hyperkeratosis) and inflammatory cell infiltrates in the dermis. Infiltrating T lymphocytes and its secreted products in psoriasis skin lesions play roles in driving and maintaining the disease. T-cells are found interspersed between keratinocytes in the epidermis and in large amount in the dermis. The psoriatic epidermis has predominantly CD8+ T cells while the dermis has a mix of CD4+ and CD8+ T cells, but mainly dominated by CD4+ T cells [2].

Psoriasis lesions develop upon epidermal influx [3] and clonal expansion of CD8+ T cells, indicating persistent CD8+ T cell recruitment and activation by locally presented autoantigens [4,5]. However, the nature of the autoantigens that drive T cell activation are only recently characterized.

Cathelicidin (LL37), a keratinocyte-derived peptide that plays a role in antimicrobial defence in damaged skin, was identified as an autoantigen for T cells in psoriasis [6]. In a new study, A Disintegrin and Metalloprotease Domain containing Thrombospondin Type 1 Motif-like 5 (ADAMTSL5), a protein important for microfibril function, was found to be produced by melanocytes and when presented by HLA-C*06:02 can be an activating antigen for IL-17-producing CD8+ T cells in psoriasis [7]. IL-17 producing T cells are considered central pathogenic immune cells in psoriasis.

ADAMTSL5 is a novel protein discovered by Tsutsui et al in the late 2000s and is described to bind to fibrillin-1 and promote fibril formation [8]. The role of ADAMTSL5 in microfibril formation is of considerable interest as a crucial mechanism for growth factor regulation in ECM [9]. Although its physiologic function is poorly defined, of particular interest is its role as an autoantigen in psoriasis. Melanocytes were recently implicated in the secretion of ADAMTSL5 by Prinz group [7]. However, there are no studies that have characterized the expression of ADAMTSL5 in human skin.

Our group studied the cutaneous expression of ADAMTSL5 by performing immunohistochemistry staining on lesional and nonlesional skin biopsies from psoriasis patients using three different commercially available ADAMTSL5 antibodies. Our results showed a strong expression of ADAMTSL5 in keratinocytes, some dermal cells and blood vessels in addition to melanocytes. The pattern of ADAMTSL5 expression is thus similar to the infiltrating pattern of T-cells and dendritic cells in psoriasis.

## Materials and Methods

### Immunohistochemistry (IHC) and Immunofluorescence (IF)

Standard procedures were used for IHC and IF as previously described [10]. For IHC, non-lesional and lesional frozen tissue sections from psoriasis patients were stained with ADAMTSL5 made in rabbit (Cat #NBP1-93438) (Novus Biologicals, Littleton, CO), ADAMTSL5 made in goat (sc-241781)(Santa Cruz Biotechnology, Dallas, TX), and ADAMTSL5 made in chicken (SAB3500142) (Sigma-Aldrich, Saint Louis, MO). Biotin-labeled goat anti-rabbit, rabbit anti-goat, and goat anti-chicken (Vector Laboratories, Burlingame, CA) were used to detect the antibodies, respectively. The staining signal was amplified with avidin-biotin complex (Vector Laboratories, Burlingame, CA) and developed using chromogen 3-amino-9-ethylcarbazole (Sigma-Aldrich, St. Louis, MO).

For double immunofluorescence, lesional frozen sections from psoriasis patients were fixed with acetone. The tissue sections were initially blocked with 10% normal chicken serum (Vector Laboratories) for the goat antibody and 10% normal goat serum (Vector Laboratories) for chicken and rabbit antibodies, respectively.

Different tissue sections were then incubated with ADAMTSL5 (goat, chicken and rabbit) antibodies together with MELAN A (mouse) antibody (Novocastra, New Castle, UK) overnight at 4°C. The following day, the tissue sections were amplified with ALEXA FLUOR 546 goat anti-rabbit (Invitrogen, Eugene, OR), ALEXA FLUOR 568 goat anti-chicken (Life Technologies, Eugene, OR), ALEXA FLUOR 594 chicken anti-goat (Invitrogen, Eugene, OR), and ALEXA FLUOR 488 goat anti-mouse (Life Technologies, Eugene, OR) for 30 min.

Images were acquired using the appropriate filters of a Zeiss Axioplan 2 widefield fluorescence microscope with a Plan Neofluar 20 × 0.7 numerical aperture lenses and a Hamamatsu Orca ERcooled charge–coupled device camera, controlled by METAVUE software (MDS Analytical Technologies, Downington, PA, USA).

Images in each figure are presented both as single color stains (green and red) located above the merged image, so that localization of two markers on similar or different cells can be appreciated. Cells that co-express the two markers in a similar location are yellow in color. A white line denotes the junction between the epidermis and the dermis.

## Results

### Distribution of ADAMTSL5 in nonlesional and lesional psoriatic skin

**Figure 1:**
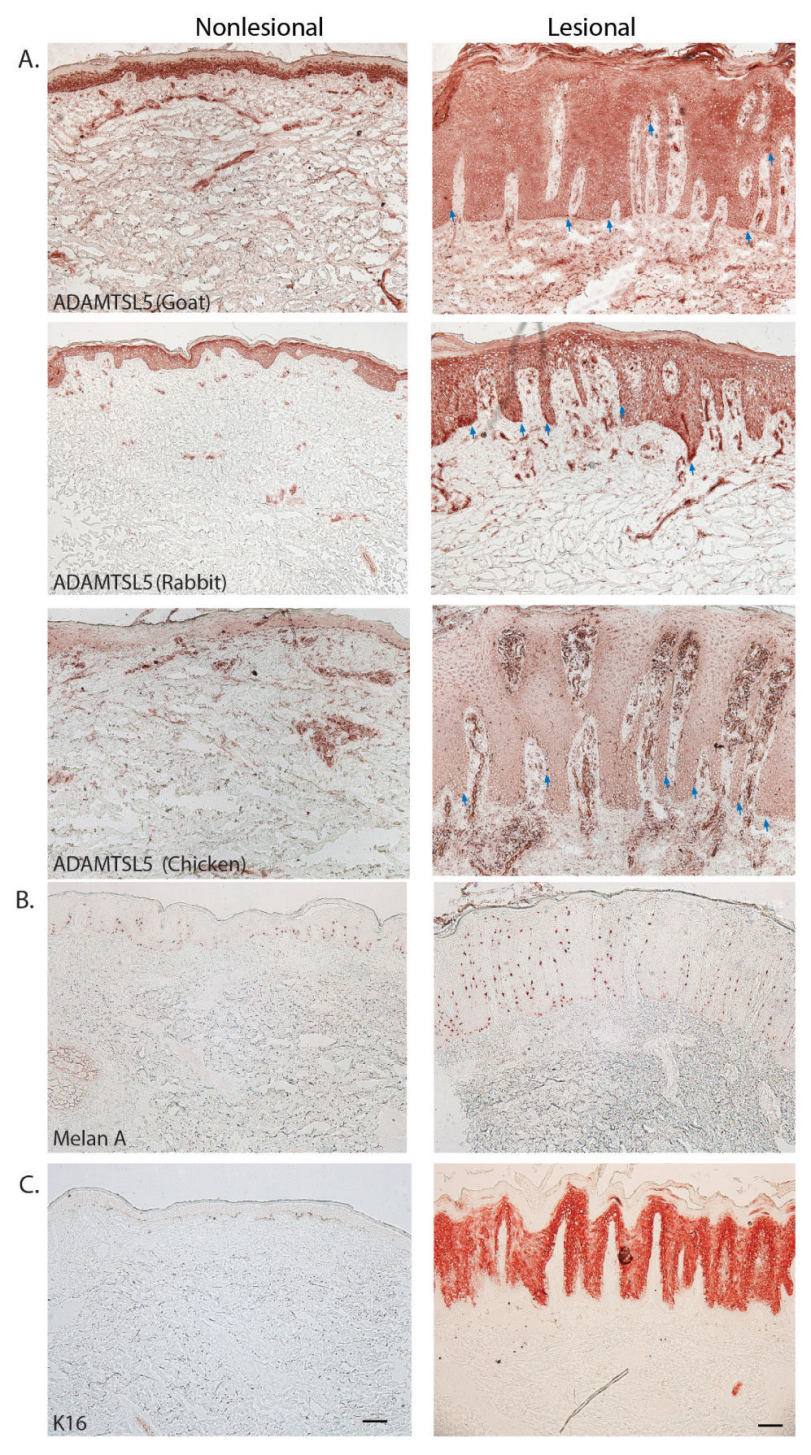
Distribution of ADAMTSL5 in nonlesional and lesional psoriatic skin Representative images for immunohistochemistry staining of ADAMTSL5, Melan-A, and K16 in psoriasis nonlesional and lesional skin. There is widespread distribution and expression of ADAMTSL5 raised in goat, rabbit, and chicken in keratinocytes, melanocytes, blood vessels and dermal cells (A). The staining pattern is similar to melanocytes (Melan A) in the basal layer of the epidermis (B) and keratinocytes stained by K16 (C). The blue arrows indicate ADAMTSL5 expression in melanocytes in lesional psoriatic skin. Size bar = 100um.

Immunohistochemistry staining of ADAMTSL5 was done on non-lesional and lesional psoriasis tissue using 3 different antibodies. Representative images are shown in Figure 1A. All antibodies reacted to scattered basal cells in the epidermis with the morphology of melanocytes (arrows) similar to Melan A staining of melanocytes in Figure 1B.

The ADAMTSL5 antibodies made in rabbit and goat showed a strong expression in keratinocytes throughout the epidermis similar to K16 staining in Figure 1C, while the ADAMTSL5 antibody made in chicken showed a weaker expression in keratinocytes. All antibodies also showed positive expression on some dermal cells and blood vessels.

Images in higher magnification together with respective negative controls are shown in Supplemental Figure 1.

**Figure 2:**
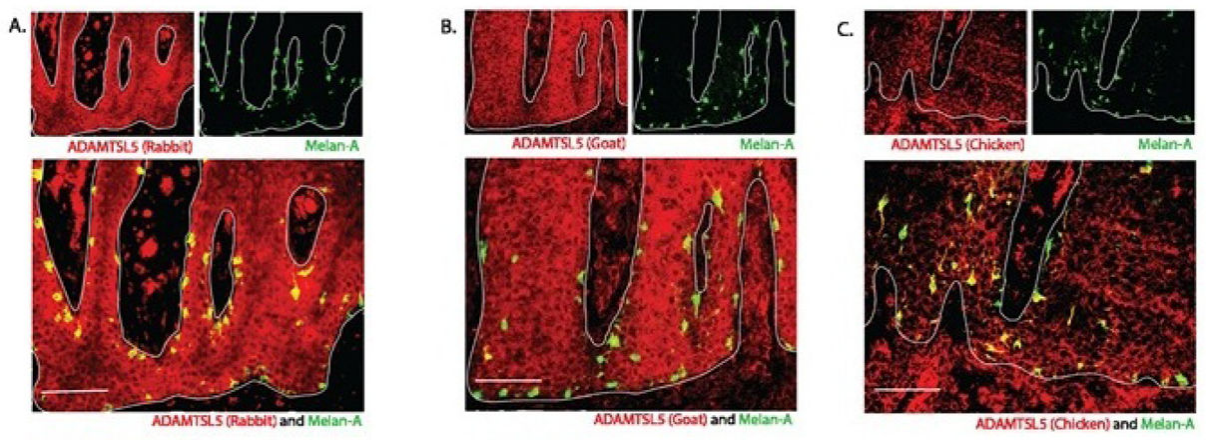
ADAMTSL5 distribution and co-localization with melanocytes. Representative double immunofluorescence images of ADAMTSL5 made in rabbit (A), goat (B), and chicken (C) in psoriasis lesional skin confirming expression of ADAMTSL5 in keratinocytes and co-localization of ADAMTSL5 with melanocytes (Melan-A). Cells that co-express the two markers in a similar location are yellow in color. White line denotes the junction between epidermis and the dermis. Size bar = 100um.

We performed double immunofluorescence staining on ADAMTSL5 (goat, rabbit, and chicken) and Melan A to confirm the co-expression of ADAMTSL5 with melanocytes (Figure 2). We observed that many ADAMTSL5+ cells (in red) co-localized with melanocytes (in green) in the basal layer of the epidermis. The staining also confirmed the expression of ADAMTSL5 in epidermal keratinocytes.

### ADAMTSL5 showed similar distribution with T-cells and DCs in Psoriasis

To show the characteristic staining pattern and distribution of ADAMTSL5 in psoriasis, we performed immunohistochemistry staining of ADAMTSL5, T-cells (CD3) and dendritic cells (CD11c) on lesional and non-lesional tissue from psoriasis patients.

The ADAMTSL5 antibody made in chicken showed distinct expression in dermal cells surrounding blood vessels (perivascular) and near the dermo-epidermal junction (blue arrows) which is similar to the pattern and distribution of T-cells and dendritic cells in lesional and non-lesional psoriatic skin.

**Figure 3:**
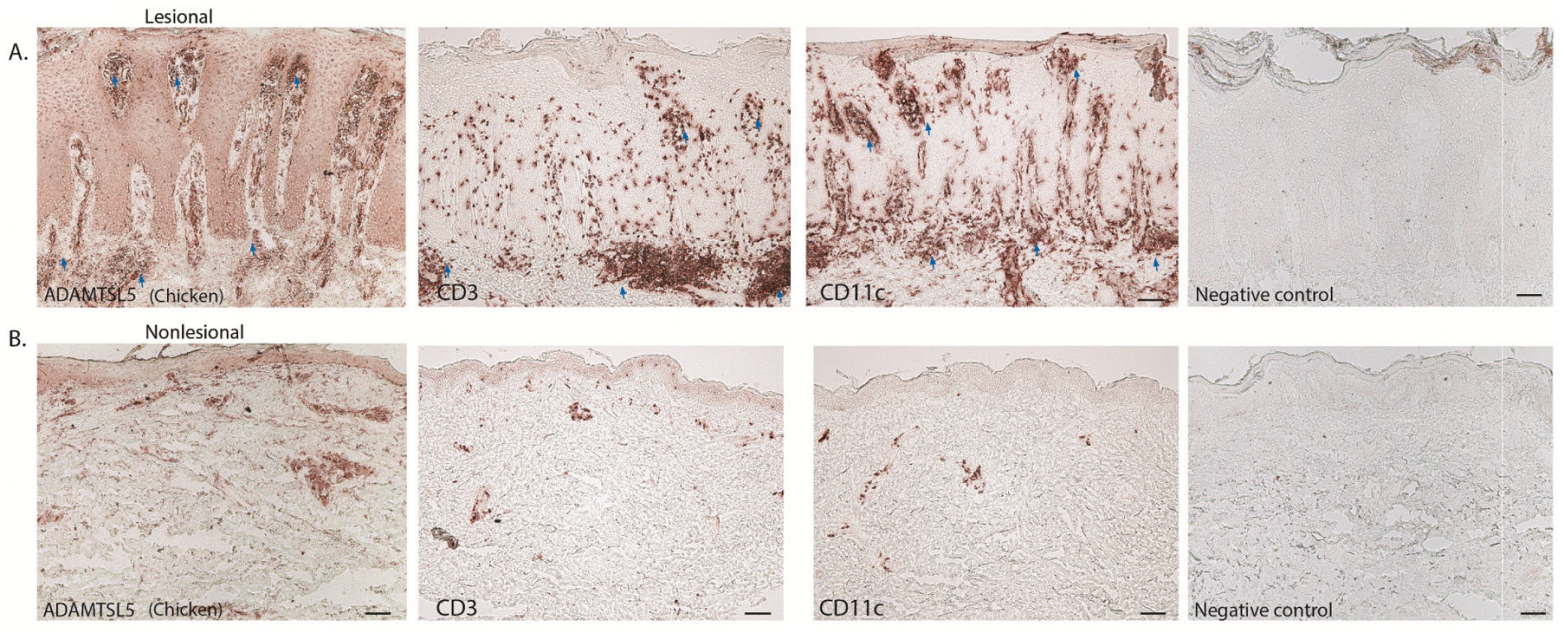
ADAMTSL5 staining pattern is similar to T-cells and DCs. Representative images of ADAMTSL5 expression in psoriasis lesional (A) and nonlesional (B) tissue showing distinct dermal cells and large aggregates in the dermis (blue arrows), in areas that match the pattern of infiltrating T cells (CD3) and dendritic cells (CD11c), with the corresponding negative controls. Size bar = 100um.

Consistent with previous studies, T-cells are present in both the epidermis and the dermis and are significantly increased in lesional skin with a characteristic pattern of aggregates in the dermis. On the other hand, dendritic cells are widely distributed in the epidermis and dermis. Most of these dendritic cells are located near the dermo-epidermal junction and in the papillary dermis. The negative controls did not show any staining (Figure 3).

## Discussion

ADAMTSL5 is a novel and intriguing fibrillin-1-, fibrillin-2- and heparin-binding member of the ADAMTS superfamily. Bader et al have shown that it does not appear to accelerate microfibril biogenesis the way ADAMTSL4 and ADAMTSL6 were shown to do in vitro and in vivo, respectively [11–13]. The effect of ADAMTSL5 may be modification of the action of fibrillin-1 on microfibril biogenesis by a competitive mechanism [11]. During late mouse embryogenesis, ADAMTSL5 may be distributed on epithelial and connective tissue of organs. The protein was observed to be localized in the pericellular and subcellular ECM of cells [11].

The study of the ADAMTS field spans only 2 decades. It has become known through analysis of spontaneous mutations in humans and animals and genetically engineered animals. Although they are related to A disintegrin and metalloprotease (ADAM) because of the similarity of their metalloproteinase domain to that of snake venom metalloproteinase, ADAM is membrane-anchored and ADAMTS is secreted protease.

There are 19 ADAMTS and 7 ADAMTSL each of which emerges from a distinct gene [14,15]. ADAMTS are known to process collagen as pro-collagen N-proteinase, cleave matrix proteoglycans, inhibit angiogenesis, and cleave von Willebrand factor for blood coagulation homeostasis [15]. Understanding genetic mutations in ADAMTSL gives us an insight into the role of this protein. ADAMTSL2 mutations cause human geleophysic dysplasia characterized by short stature, small hands and feet, stiff joints, and thick skin. This may be a result of increased TGFβ activity due to the loss of interaction between the ADAMTSL2, latent TGFβ-binding protein-1 (LTBP-1) and fibrillin-1 (FBN1) [16,17].

ADAMTSL4 mutations cause isolated ectopia lentis (dislocation of the ocular lens) due to impaired fibrillin-1 rich suspensory ligament of the lens [18]. Here, it is important to note that the functions of ADAMTSL are tissue-specific in organs with fibrillin-1 [8]. ADAMTSL2, ADAMTSL4, and ADAMTSL6 bind FBN1 [12,13,17] accelerate the formation of fibrillin-1 microfibrils [12,13,19], and may regulate TGFβ.

A recent study by Prinz’s group implicates ADAMTSL5 as an auto antigen in psoriasis, which has been categorized as an autoimmune disease. They have identified its expression associated with melanocytes and as an activating antigen of IL-17-producing T cells in HLA-C*06:02 positive patients. The T cell expansion juxtaposed to the melanocytes does not cause cytotoxicity, rather, an increase in melanocytes in psoriasis consistent with its key feature of epidermal hyperplasia [7].

We examined the expression of ADAMTSL5 in psoriasis skin and confirmed the staining of scattered basal cells with melanocyte morphology, in agreement with the observations of Prinz’s group. However, there was also a strong staining of keratinocytes and some dermal cells using the antibodies made in goat and rabbit. ADAMTSL5 antibody made in chicken showed a strong expression on perivascular dermal cells and dendritic cells, but weaker staining on keratinocytes in psoriasis lesional skin. Because T cell infiltrates in psoriasis are interspersed throughout the epidermis and form very large dermal infiltrates, there is a potential disconnect between the basal cell location of melanocytes and T cell infiltrates. If the immune reaction followed melanocyte expression of ADAMTSL5, we would expect T-cells to be more junctional rather than the epidermal and dermal patterns in psoriasis. The strong ADAMTSL5 staining on keratinocytes could result from its synthesis in keratinocytes or, alternatively, from deposition of the protein in keratinocytes following melanosome transfer from melanocytes to keratinocytes, as part of the normal pigmentation pathway. As an intracellular antigen in keratinocytes or dermal connective tissue cells, ADAMTSL5 might trigger CD8+ T-cells (Tc17) reactivity to multiple cell types expressing the antigen in the context of HLA-C*06:02. On the other hand, extracellular deposition of ADAMTSL5, as observed in development [11], could also be responsible for stimulating antigen specific CD4+ T-cells (Th17 T-cells) that are also seen in psoriasis.

## Supplementary Material

Suppl Figures
