# Supplementary material for: Cutaneous Expression of A Disintegrin-like and Metalloprotease domain containing Thrombospondin Type 1 motif-like 5 (ADAMTSL5) in Psoriasis goes beyond Melanocytes
Source: J Pigment Disord. Author manuscript; Available in PMC 2016 Nov 15. (PMC5110039; doi:10.4172/2376-0427.1000244)
Supplement: Suppl Figures [file NIHMS824679-supplement-Suppl_Figures.pdf]

Supplemental Figure 1

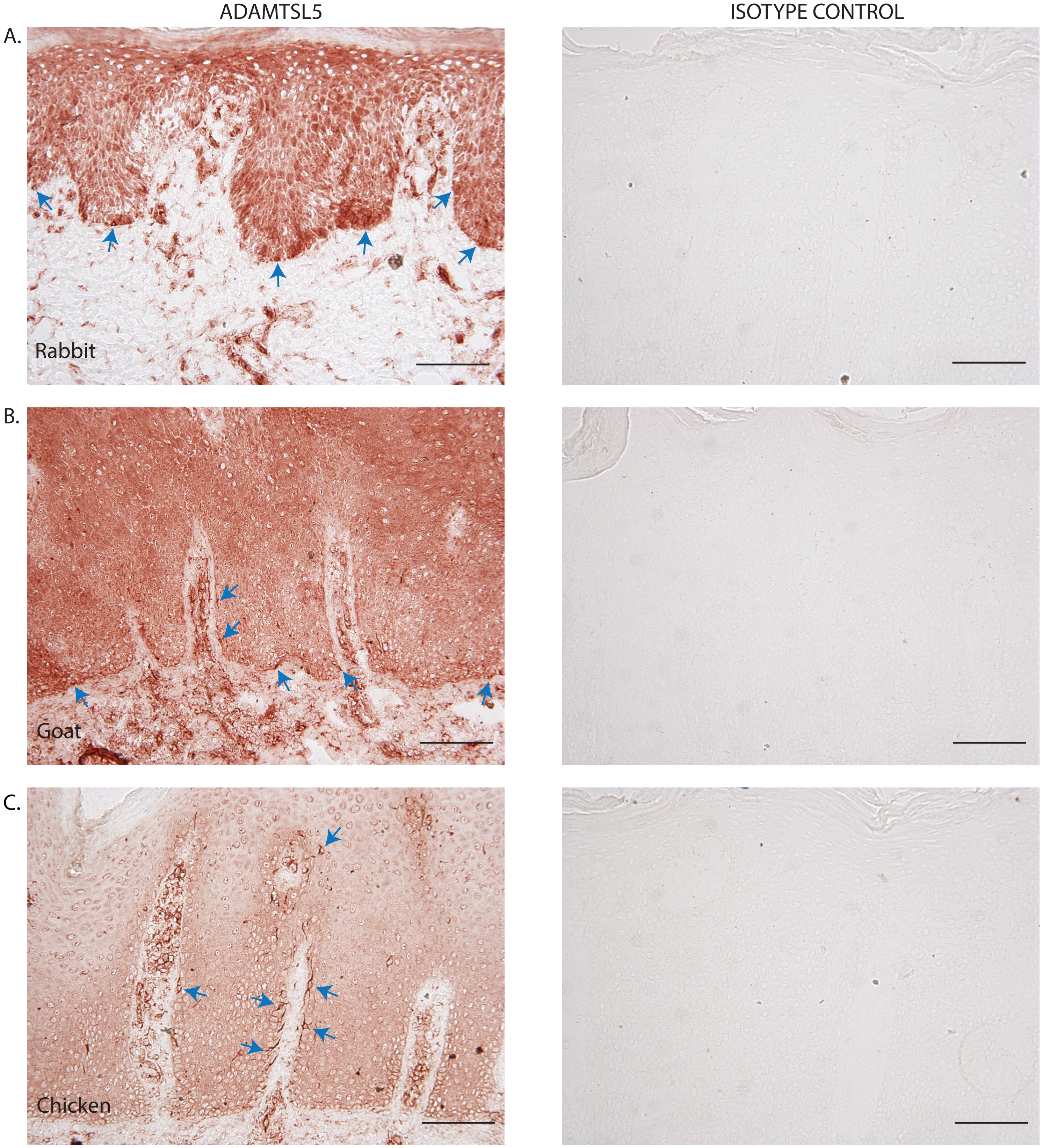

Representative images of ADAMTSL5 made in rabbit (A), goat (B), and chicken (C) on psoriasis lesional skin in higher magnification show expression on melanocytes and on epidermal keratinocytes with different intensities. Isotype controls show negative reactivity.
